# Cost-effectiveness analysis of amivantamab plus chemotherapy versus chemotherapy alone in NSCLC with EGFR Exon 20 insertions

**DOI:** 10.3389/fonc.2024.1368804

**Published:** 2024-03-22

**Authors:** Ping Yue, Mengwei Zhang, Yuanying Feng, Yuan Gao, Chao Sun, Peng Chen

**Affiliations:** ^1^ Department of Thoracic Oncology, Lung Cancer Diagnosis and Treatment Center, Tianjin Medical University Cancer Institute and Hospital, Tianjin, China; ^2^ National Clinical Research Center for Cancer, National Key Laboratory of Druggability Evaluation and Systematic Translational Medicine, Tianjin’s Clinical Research Center for Cancer, Tianjin, China; ^3^ Department of General Practice, Ruijin Hospital, Shanghai Jiao Tong University School of Medicine, Shanghai, China

**Keywords:** amivantamab, chemotherapy, cost-effectiveness analysis, non-small-cell lung cancer, papillon

## Abstract

**Objective:**

Amivantamab plus chemotherapy has been proved to be an efficient treatment strategy for non–small-cell lung cancer (NSCLC) with epidermal growth factor receptor (EGFR) exon 20 insertions. The aim of this study was to conduct the cost-effectiveness analysis of amivantamab-chemotherapy compared with chemotherapy alone in NSCLC harboring EGFR exon 20 insertion mutations.

**Methods:**

We constructed a Markov model based on the data derived from the PAPILLON trial. We evaluated the cost, quality-adjusted life years (QALYs), and incremental cost-effectiveness ratio (ICER). One-way and probabilistic sensitivity analyses were used to evaluate the influence of different parameters on this model.

**Results:**

Compared with chemotherapy alone, amivantamab combined with chemotherapy treatment gained an incremental effectiveness of 0.473 QALYs and an incremental cost of $361,950.952, which resulted in an ICER of $765,224/QALY. The ICER was much higher than the willingness-to-pay threshold of 15,0000/QALY. One-way sensitivity analysis revealed that amivantamab cost was the leading influential factor in the model.

**Conclusions:**

Compared with chemotherapy alone, amivantamab plus chemotherapy is not a cost-effective first-line treatment choice for NSCLC patients with EGFR exon 20 insertions. The costly price of amivantamab is one of the major reasons for the high cost of this combined treatment strategy. Therefore, it is imperative to take into account the high cost of amivantamab in the subsequent clinical application and strive to attain a relative equilibrium between its significant clinical benefit and economic encumbrance.

## 1 Introduction

Cancer is a prominent contributor to global mortality rates, which has become a heavy burden for physical, emotional, and financial strain on individuals, communities, and entire health systems (1). The emergence of numerous novel drugs improves the survival outcomes of cancer patients, particularly those with lung cancer. Nevertheless, the price of new drugs is usually too high to afford for individuals, which also poses a tremendous strain on healthcare systems, especially in low- and middle-income countries. Notably, lung cancer is the main cause of cancer-related death around the world, and non–small-cell lung cancer (NSCLC) accounts for 85% of lung cancers (2).

Epidermal growth factor receptor (EGFR) is the most prevalent activating mutation in NSCLC (3). Among the EGFR mutations, exon 19 mutations are the most frequent mutation, followed by exon 21 mutations, and exon 20 insertions (4, 5). NSCLC harboring EGFR exon 20 insertion mutations do not respond well to tyrosine kinase inhibitors (TKIs) because of the existence of conformational change at the kinase-active site (6). Thus, platinum-based chemotherapy is recommended as first-line therapy for NSCLC harboring EGFR exon 20 insertion mutations rather than EGFR-TKIs (7). Amivantamab, a bispecific antibody that targets both the EGFR and Mesenchymal-Epithelial Transition (MET) factor, has been demonstrated to have antitumor activity with multiple mechanisms proved by a series of basic research (8, 9). The combined effects of these mechanisms allow it to overcome ligand-site resistance to TKIs in NSCLC patients with EGFR exon 20 insertions.

PAPILLON trial is a phase 3, international, randomized trial designed to evaluate the efficacy and safety of amivantamab plus carboplatin–pemetrexed (amivantamab–chemotherapy) against standard chemotherapy as a first-line regimen for patients diagnosed with advanced NSCLC harboring EGFR exon 20 insertion mutations. The trial revealed that amivantamab–chemotherapy significantly prolonged the progression-free survival (PFS) compared with chemotherapy alone (11.6 *vs*. 6.7 months; HR, 0.40; 95% CI 0.30–0.53; *p*<0.001). Most side effects associated with amivantamab were mild. Hence, amivantamab–chemotherapy was an option for an effective first-line regimen in patients with advanced NSCLC harboring EGFR exon 20 insertion mutations (10).

Despite the clinical advancements achieved by amivantamab–chemotherapy in the field of advanced NSCLC harboring EGFR exon 20 insertion mutations, the high cost of amivantamab has raised concerns about its affordability and economic feasibility. Consequently, its cost-effectiveness in treating advanced NSCLC harboring EGFR exon 20 insertion mutations requires further investigation. Our study aims to assess the cost-effectiveness of amivantamab–chemotherapy compared with chemotherapy alone in treating advanced NSCLC harboring EGFR exon 20 insertion mutations.

## 2 Materials and methods

### 2.1 Patients and intervention

The enrolled patients were all derived from the PAPILLON trial. NSCLC patients harboring EGFR insertion mutations in EGFR exon 20 who were over 18 years old and had not received any prior treatment were recruited. Patients were randomly divided into two groups in a 1:1 ratio to receive amivantamab plus chemotherapy (amivantamab–chemotherapy) or chemotherapy alone. A total of 308 patients were enrolled, 153 received amivantamab–chemotherapy and 155 received chemotherapy alone. In the chemotherapy group, patients received carboplatin at a targeted area under the concentration-time curve of 5 mg/mL/min for up to 4 cycles and received pemetrexed at a dose of 500 mg/m^2^ of body surface area until disease progression. In the amivantamab–chemotherapy group, patients received a dose of 1400 mg amivantamab (1750 mg for weight ≥80 kg) per week for the first 4 weeks and then received a dose of 1750 mg amivantamab (2100 mg for weight ≥80 kg) every 3 weeks until disease progression.

In our study, we assumed that the patients with a baseline weight of 70 kg, body surface area of 1.86 m^2^, and a creatinine clearance of 70 mL/min as standard (11). Only when adverse events (AEs) ≥ grade 3 and incidence rate ≥5% in either the amivantamab–chemotherapy or chemotherapy group, AEs were identified for analysis. This study was carried out based on the PAPILLON study and did not involve other human participants. Hence, there is no need for the approval of the independent ethics committee.

### 2.2 Model construction

**Figure 1:**
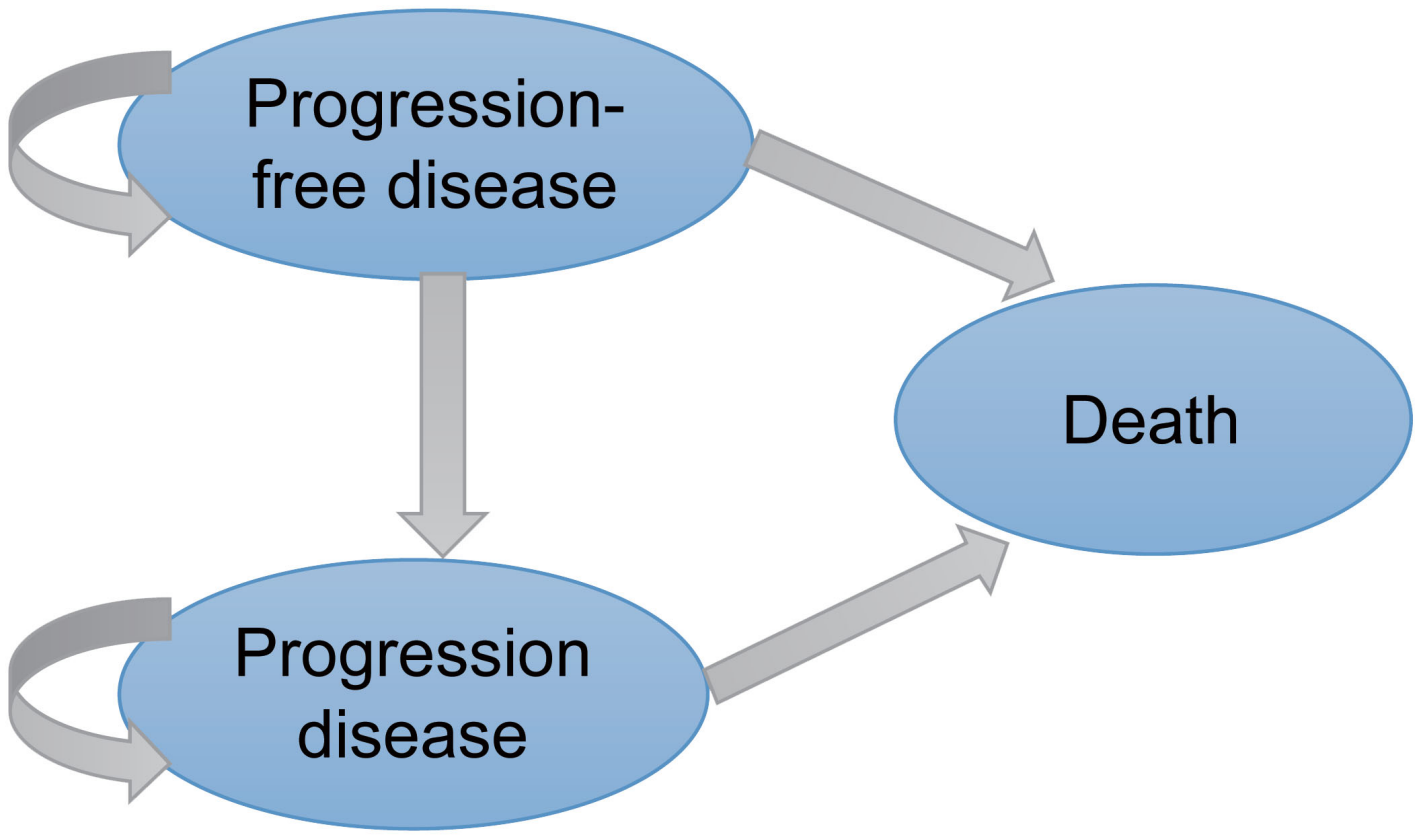
Model Construction.

**Figure 2:**
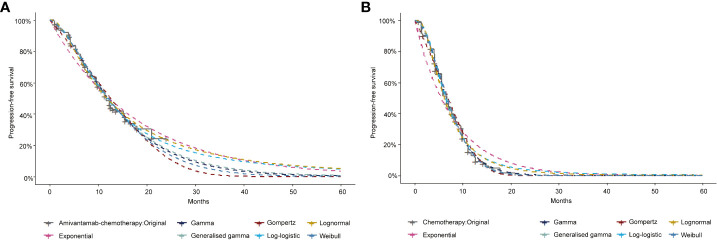
The exploration and fitting of progression-free survival curves in Amivantamab–chemotherapy **(A)** and Chemotherapy **(B)**.

A three-stage Markov model was established by the TreeAge Pro 2022 software, including PFS, progressive disease (PD), and death (**Figure 1**). We extended the simulation period to 5 years because the median overall survival (OS) of amivantamab–chemotherapy has not been reached in the PAPILLON trial. The simulated PFS and OS fitted with different distributions were displayed in **Figure 2** and **Supplementary Figure 1**. The simulation cycle duration was 3 weeks, which was consistent with the PAPILLON trial.

In this study, a 3% annual discount rate was applied for future health utility and cost (12). The analysis included three key parameters: total cost, quality-adjusted life years (QALYs), and incremental cost-effectiveness ratio (ICER). A willingness-to-pay (WTP) threshold of $150,000/QALY was set to evaluate whether the treatment represented a cost-effective choice (13). A treatment was considered cost-effective if its calculated ICER was lower than this established threshold.

### 2.3 Model survival and transition probabilities

The OS and PFS data of amivantamab–chemotherapy and chemotherapy were extracted by the GetData Graph Digitizer. The estimates of the OS curve and PFS curve for both groups were reconstructed by R software. A series of statistical distributions, such as the Log-logistic, Log-normal, Weibull, Gompertz, Exponential, and Gamma distributions were utilized to fit the pseudo-individual patient data set. This study chose the most suitable survival function with the lowest Akaike information criterion (AIC) and Bayesian information criterion values (BIC) (14). Detailed model diagnostics were presented in **Supplementary Tables 1** and **2**. The shape parameter (γ) and the scale parameter (λ) of the suitable function were gained from R software. The transition probabilities were calculated based on S(t)=1/(1+λt^γ^) (15).

### 2.4 Cost and utility estimates

This study focused on direct medical costs, including drug costs, subsequent treatment, routine follow-up, and costs associated with AEs. The average drug price was sourced from the US Centers for Medicare & Medicaid Services (January 2024 ASP Drug Pricing). The cost of AEs, follow-up, and subsequent treatment were extracted from previous research (16–19).

QALYs were used to assess the effectiveness of each health condition. The utilities of PFS and PD in our model were 0.71 and 0.67 (20). To evaluate the negative effect of AEs, the disutility of AEs was also taken into account in this model (17, 21). The detailed costs and utilities were all displayed in **Table 1**.

**Table 1:** Model parameters and distributions.

Variable	Base value	Range	Distribution
Drug cost			
Amivantamab per cycle (2mg)	19.975	15.980-23.970	Gamma
Pemetrexed (10mg)	10.554	8.435-12.665	Gamma
Carboplatin (50mg)	3.599	2.879-4.319	Gamma
Grade ≥3 AEs incidence in Amivantamab-chemotherapy			
Neutropenia	0.33	–	Beta (10)
Paronychia	0.07	–	Beta (10)
Rash	0.11	–	Beta (10)
Anemia	0.11	–	Beta (10)
Leukopenia	0.11	–	Beta (10)
Thrombocytopenia	0.10	–	Beta (10)
Hypokalemia	0.09	–	Beta (10)
Asthenia	0.05	–	Beta (10)
Grade ≥3 AEs incidence in Chemotherapy			
Neutropenia	0.23	–	Beta (10)
Paronychia	0	–	Beta (16)
Rash	0	–	Beta (10)
Anemia	0.12	–	Beta (10)
Leukopenia	0.03	–	Beta (10)
Thrombocytopenia	0.10	–	Beta (10)
Hypokalemia	0.01	–	Beta (10)
Asthenia	0.03	–	Beta (10)
Cost of adverse event per cycle			
Neutropenia	454.26	363.41-545.11	Gamma (18)
Paronychia	9396.00	7516.80-11275.20	Gamma (19)
Rash	400.00	320.00-480.00	assumption
Anemia	336.63	269.30-403.95	Gamma (18)
Leukopenia	454.26	363.41-545.11	Gamma (18)
Thrombocytopenia	1640.00	1312.00-1968.00	Gamma (18)
Hypokalemia	3000.00	2400.00-3600.00	Gamma (17)
Asthenia	113.59	90.87-136.31	Gamma (21)
Utility			
Utility of progression-free	0.71	0.57-0.85	Beta (20)
Utility of progressed-disease	0.67	0.47-0.71	Beta (20)
Disutility of neutropenia	0.20	0.16-0.24	Beta (21)
Disutility of paronychia	0.1	0.08-0.12	assumption
Disutility of rash	0.03	0.02-0.04	Beta (16)
Disutility of anemia	0.07	0.06-0.08	Beta (21)
Disutility of leukopenia	0.2	0.16-0.24	Beta (21)
Disutility of thrombocytopenia	0.11	0.09-0.13	Beta (21)
Disutility of hypokalemia	0.03	0.02-0.04	Beta (17)
Disutility of asthenia	0.07	0.06-0.08	Beta (21)
Other			
Subsequent therapy cost per cycle	1858.00	1486.40-2229.60	Gamma (11)
Follow-up cost per cycle	118.39	94.71-142.07	Gamma (11)
Body surface area	1.86	1.49-2.23	Beta (11)

### 2.5 Sensitive analysis

One-way sensitivity was utilized to evaluate the uncertainty of this model. In this analysis, we introduced a 20% variation in parameter values to assess the extent of its impact on the calculated ICER value. The outcomes were displayed in the tornado diagram, which visually showed the influence of each parameter on the ICER.

We conducted probabilistic sensitivity analysis to investigate the robustness of this model. We performed 1,000 Monte Carlo simulations with key parameters. The cost parameter was described by gamma distribution and the utility parameter was characterized by beta distribution. The cost-effectiveness acceptability curves and scatterplots illustrated the likelihood of amivantamab–chemotherapy being cost-effective across the WTP threshold.

## 3 Results

### 3.1 Base case results

The baseline findings revealed that amivantamab–chemotherapy resulted in 1.904 QALYs (2.973 LYs), whereas chemotherapy alone generated 1.423 QALYs (2.161 LYs). The cost of amivantamab–chemotherapy was $430,980.857, while the cost of chemotherapy alone was $62,908.322. Based on these results, the ICER value of amivantamab–chemotherapy was $765,223.566/QALY ($453,291.299/LY) in comparison to chemotherapy alone, which exceeded the assumed WTP of $150,000/QALY (**Table 2**).

**Table 2:** Base case results of the model.

Parameters	Amivantamab–chemotherapy	Chemotherapy
LYs	2.973	2.161
QALYs	1.904	1.423
Costs ($)	430,980.857	62,908.322
ICER ($/LY)	453,291.299	–
ICER ($/QALY)	765,223.566	–

LYs, life years; QALYs, quality-adjusted life years; ICER, incremental cost-effectiveness ratio.

### 3.2 Sensitivity analysis results

**Figure 3:**
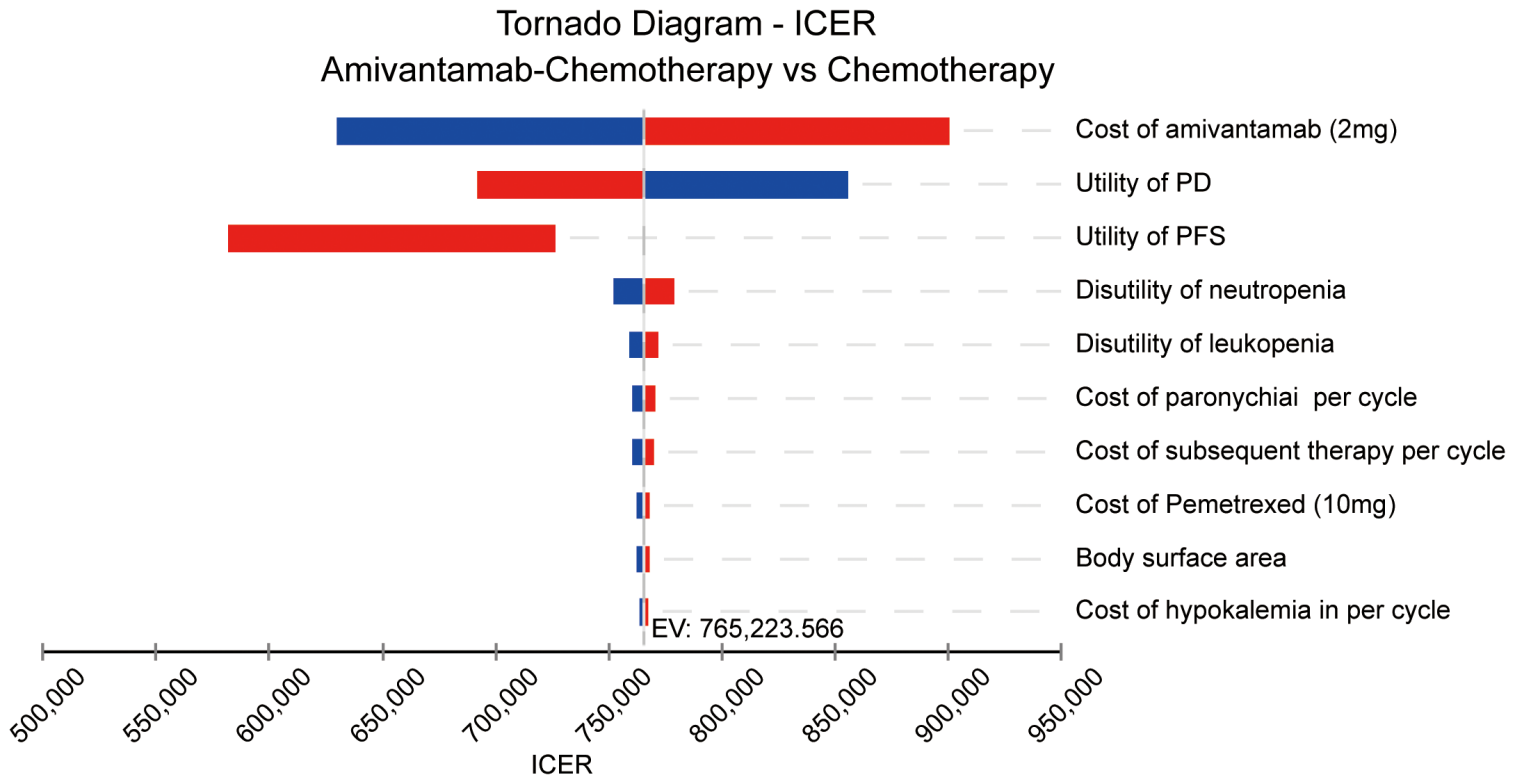
The result of the one-way sensitivity analysis. ICER, incremental cost-effectiveness ratio; PD, progressive disease; PFS, progression-free survival; EV, expected value.

The Tornado diagram of the one-way sensitivity analysis displayed the top 10 influential parameters. The price of amivantamab (2 mg) was the leading influencing factor on the results, closely followed by the utility of PD, the utility of PFS, the disutility of neutropenia, and the disutility of leukopenia (**Figure 3**).

**Figure 4:**
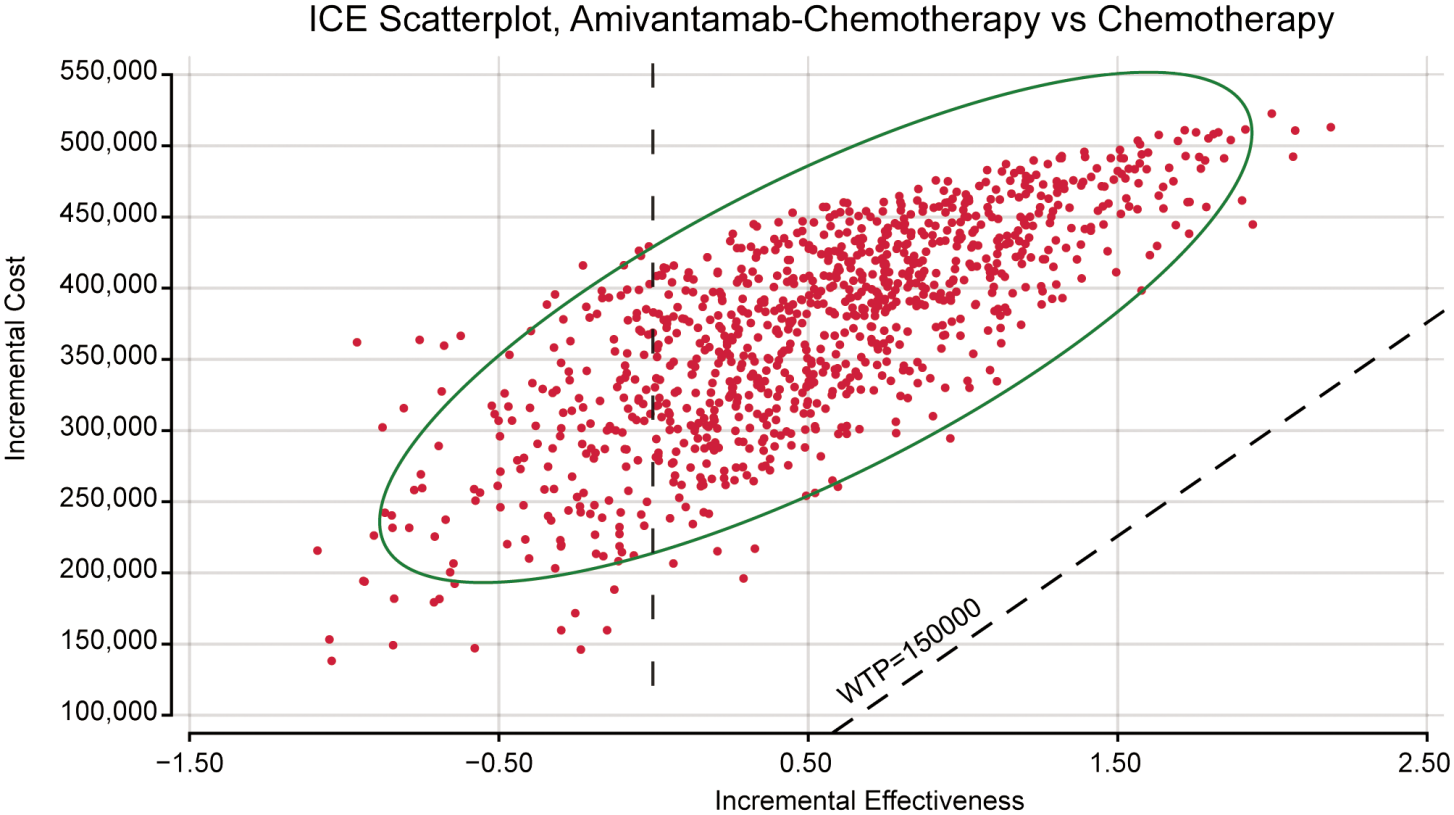
The probabilistic sensitivity analysis scatter plot. ICE, incremental cost-effectiveness; WTP, willingness-to-pay.

**Figure 5:**
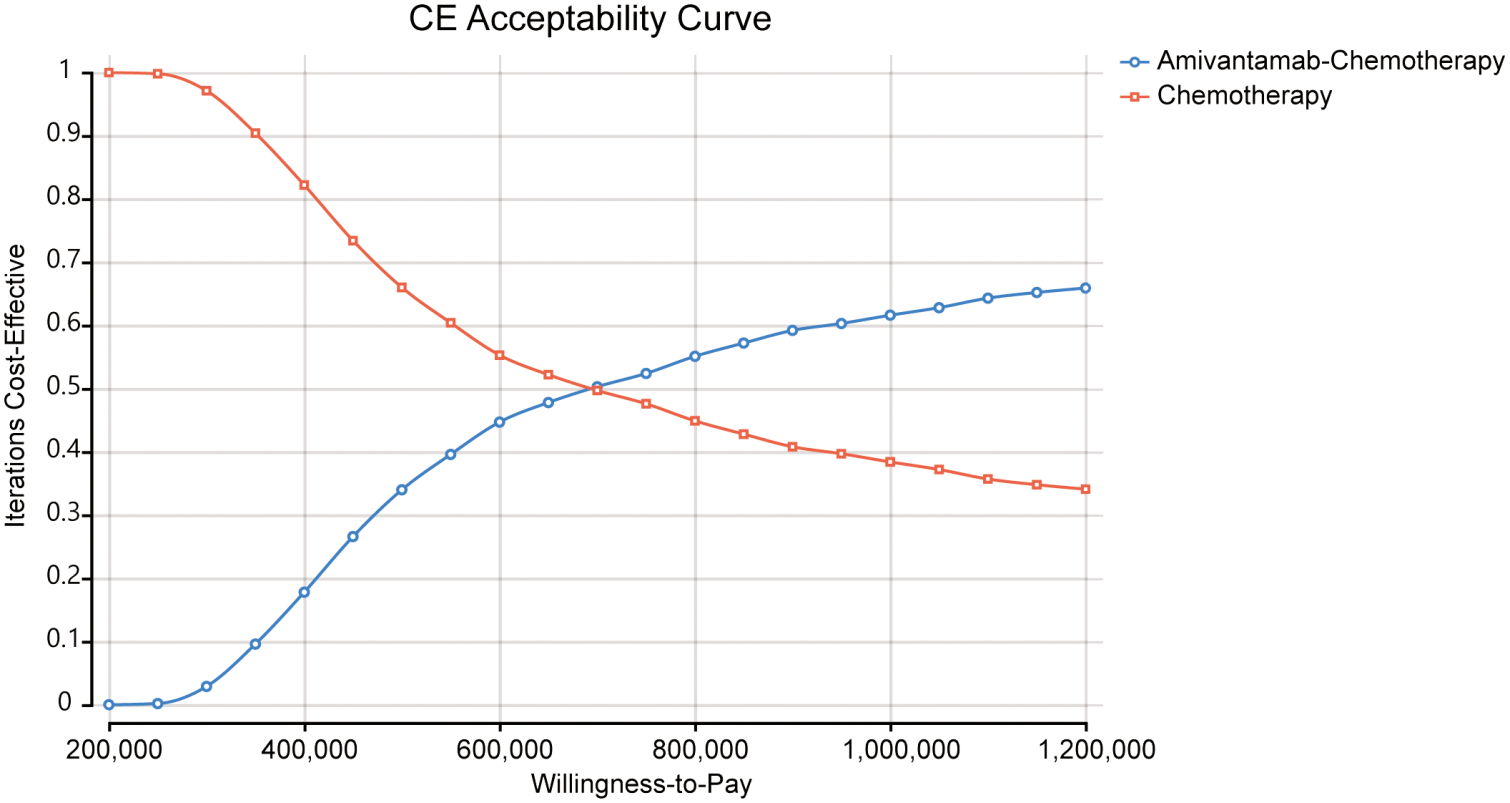
The cost-effectiveness acceptability curve.

The ICE scatter plot of probabilistic sensitivity analysis indicated that the respective points were all positioned above the WTP threshold (**Figure 4**). The cost-effectiveness acceptability curves showed amivantamab–chemotherapy was not cost-effective at all compared to the WTP threshold (**Figure 5**).

## 4 Discussions

Lung cancer is the primary cause of cancer-related death worldwide (22). The emergence of immunotherapy and targeted agents improves the survival outcomes as a whole. Among the various mutations observed in NSCLC, EGFR mutations are the most common mutations. The appearance of TKIs significantly prolongs the PFS and OS of NSCLC with EGFR mutations and its efficacy is further enhanced in the second and third generation (23). Approximately 10% of EGFR mutations involve exon 20 insertions. Regrettably, NSCLC patients harboring EGFR exon 20 insertion mutations exhibit poor response to TKIs, which restricts the application of TKIs in EGFR exon 20 insertions (24).

Amivantamab is a bispecific antibody, targeting the resistant EGFR mutations, and MET mutations and amplifications. It can simultaneously bind to the extracellular structures of EGFR and c-Met, block ligand binding to EGFR and MET, promote receptor degradation, and trigger antibody dependent cytotoxicity (25, 26). In the phase 1 CHRYSALIS trial, amivantamab was used for patients with advanced NSCLC harboring EGFR exon 20 insertion mutations. It significantly prolonged the OS and PFS of patients compared with the real-world data (27, 28). However, the efficacy and safety of amivantamab-chemotherapy were not fully investigated in the CHRYSALIS trial.

Based on the above findings, Zhou et al. designed and executed a phase 3, international, randomized PAPILLON trial to evaluate the efficacy and safety of amivantamab–chemotherapy compared with chemotherapy alone. As the results of the PAPILLON trial revealed, the PFS of amivantamab–chemotherapy was longer than chemotherapy alone in first-line therapy for patients with advanced NSCLC harboring EGFR exon 20 insertion mutations. These results support the value of amivantamab–chemotherapy as an effective first-line regimen in advanced NSCLC patients harboring EGFR exon 20 insertions. However, the high cost of amivantamab may limit its widespread application.

To our knowledge, there has been no study to assess the cost-effectiveness of amivantamab so far. Therefore, our study is the first study to evaluate the cost-effectiveness of amivantamab–chemotherapy for NSCLC harboring EGFR exon 20 insertions. In this research, amivantamab–chemotherapy resulted in an ICER of $765,223.56/QALY and the ICER was much higher than WTP of $150,000/QALY. The result indicates amivantamab–chemotherapy is less cost-effective compared with chemotherapy alone for the treatment of patients with EGFR exon 20 insertions. Based on the significant clinical benefit of amivantamab–chemotherapy in NSCLC patients harboring EGFR exon 20 insertion mutations, it would be important to reduce the cost of amivantamab or combine it with other drugs to improve its effectiveness. The cost-effectiveness analysis of amivantamab may contribute to a balance between drug costs and effectiveness, and provide new perspectives on future treatments for patients.

Given the considerable disparity in health outcomes between the two regimens, even slight variations in parameter values could significantly impact the study’s results. The sensitivity analysis found that the cost of amivantamab was the most critical parameter affecting ICER values. Additionally, it underscored the utility values of PD and PFS as the next most influential factors. This finding aligns with previous research where the role of PFS or PD status was important in determining the cost-effectiveness of cancer treatments (17, 29, 30).

Though this research has many advantages, there are still some limitations. First, the clinical data was obtained from the clinical trial rather than raw data. The OS and PFS survival curves were fitted with parametric distributions, which are beyond the follow-up period of the PAPILLON trial. This may not accurately reflect the clinical course in the real world. Second, our study only included AEs with grade ≥3 and incidence ≥ 5%. Since the grade 1/2 AEs and low incidence of serious AEs had less effect on the model. Third, drug costs, the cost of AEs, the health state utility, and the disutility of AEs were obtained from the previous research, which were not provided in the PAPILLON trial. This may lead to the appearance of bias. Last, patients in the chemotherapy group were allowed to receive amivantamab after PD, which may improve the survival outcome of the chemotherapy subgroup. In anticipation of updated clinical trial data, we plan to update this study accordingly.

## 5 Conclusions

From the perspective of the American healthcare system, the amivantamab combination with chemotherapy was not a cost-effective first-line regimen compared with chemotherapy alone for advanced NSCLC harboring EGFR exon 20 insertions. Although the emergence of amivantamab significantly improves the survival outcome of NSCLC harboring EGFR exon 20 insertions, its high costs may limit its application. Thus, it is necessary to consider the balance of the therapeutic effects and amivantamab costs in future clinical applications.

## Data availability statement

The original contributions presented in the study are included in the article/**Supplementary Material**, further inquiries can be directed to the corresponding author/s.

## Author contributions

PY: Data curation, Formal analysis, Validation, Visualization, Writing – original draft, Writing – review & editing. MZ: Data curation, Validation, Visualization, Writing – review & editing. YF: Data curation, Validation, Writing – review & editing. YG: Data curation, Validation, Writing – review & editing. CS: Conceptualization, Data curation, Formal analysis, Investigation, Methodology, Software, Validation, Visualization, Writing – original draft, Writing – review & editing. PC: Conceptualization, Funding acquisition, Validation, Writing – review & editing.
