# Supplementary material for: Cost-effectiveness analysis of amivantamab plus chemotherapy versus chemotherapy alone in NSCLC with EGFR Exon 20 insertions
Source: Front Oncol. 2024 Mar 22;14:1368804. doi: 10.3389/fonc.2024.1368804 (PMC10995216; doi:10.3389/fonc.2024.1368804)
Supplement: Supplementary file 1 [file DataSheet_1.docx]

Supplementary Material

# Supplementary Figures

**A**


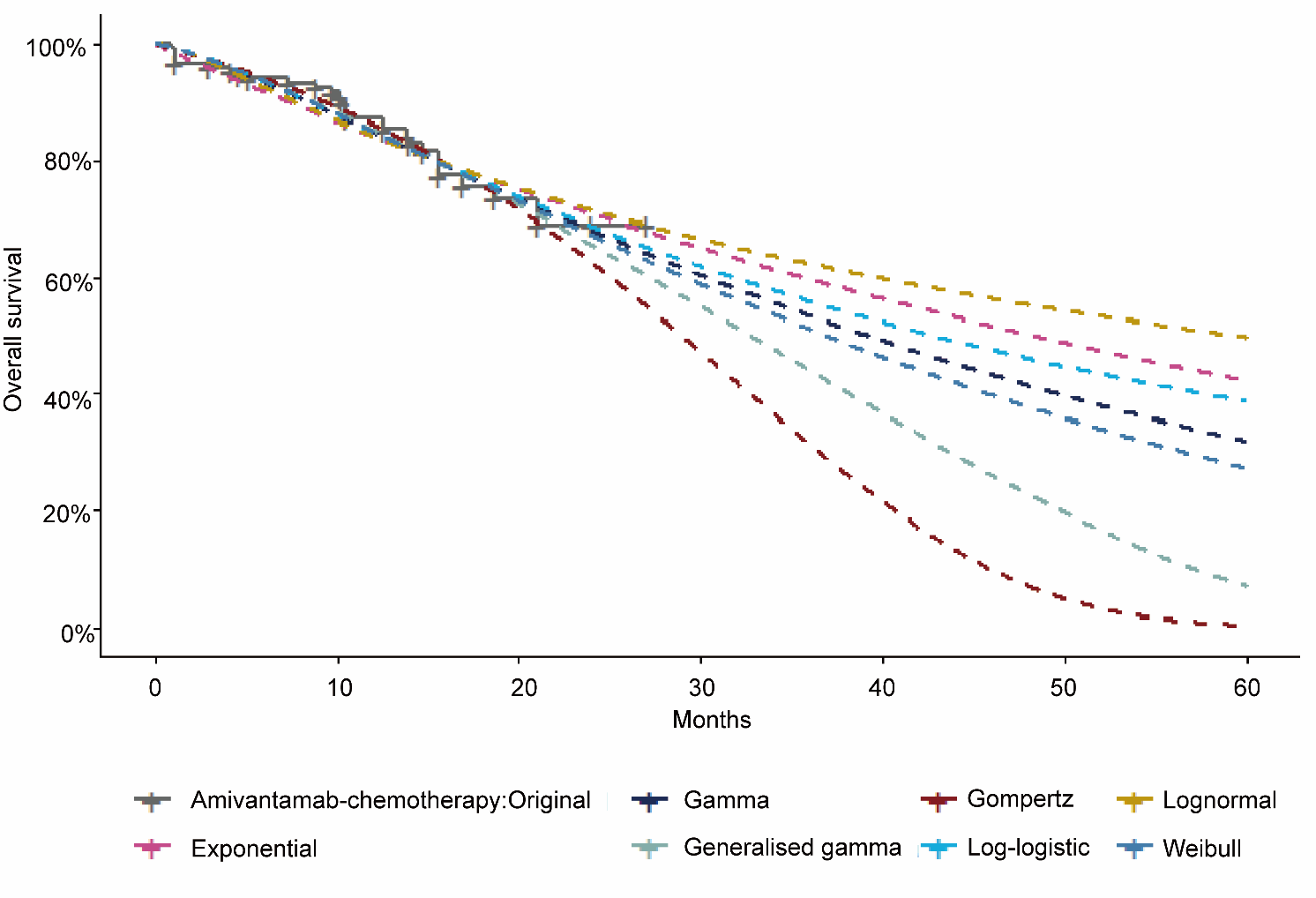


**B**


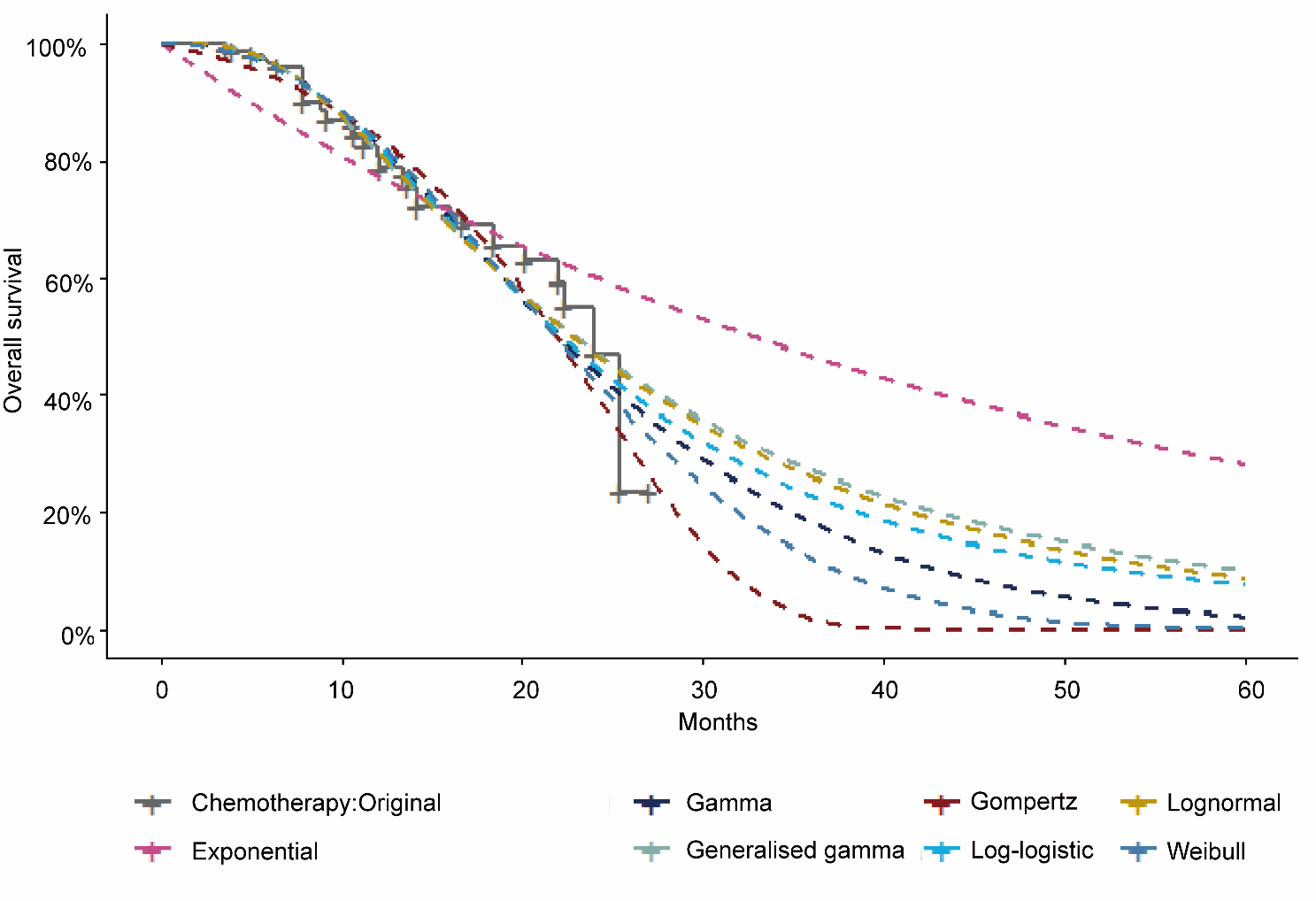


Supplementary Figure 1. The exploration and fitting of OS curves in Amivantamab–chemotherapy (A) and Chemotherapy (B).

# Supplementary Tables

**Supplementary Table 1.** Statistical fit of overall survival curves

| Distribution | Amivantamab-chemotherapy | | Chemotherapy | |
| --- | --- | --- | --- | --- |
|  | AIC | BIC | AIC | BIC |
| Exponential | 295.5088 | 298.5393 | 419.3430 | 422.3800 |
| Weibull | 295.4609 | 301.5218 | 391.2627 | 397.3366 |
| Gamma | 295.7795 | 301.8404 | 390.0351 | 396.1090 |
| Gengamma | 296.8160 | 305.9073 | 391.1636 | 400.2745 |
| Gompertz | 293.8696 | 299.9305 | 397.4517 | 403.5256 |
| Log-normal | 298.7861 | 304.8470 | 389.1810 | 395.2549 |
| Log-logistic | 296.2415 | 302.3024 | 391.0569 | 397.1308 |

AIC, Akaike Information Criteria; BIC, Bayesian Information Criteria

**Supplementary Table 2.** Statistical fit of progression-free survival curves

| Distribution | Amivantamab-chemotherapy | | Chemotherapy | |
| --- | --- | --- | --- | --- |
|  | AIC | BIC | AIC | BIC |
| Exponential | 651.6315 | 654.6619 | 812.7356 | 815.7790 |
| Weibull | 638.2422 | 644.3031 | 758.7378 | 764.8246 |
| Gamma | 637.4842 | 643.5451 | 759.0674 | 765.1543 |
| Gengamma | 639.4151 | 648.5064 | 760.3205 | 769.4507 |
| Gompertz | 644.2834 | 650.3443 | 772.5759 | 778.6628 |
| Log-normal | 640.6265 | 646.6874 | 769.0972 | 775.1841 |
| Log-logistic | 637.5487 | 643.6095 | 768.6884 | 774.7752 |

AIC, Akaike Information Criteria; BIC, Bayesian Information Criteria

**
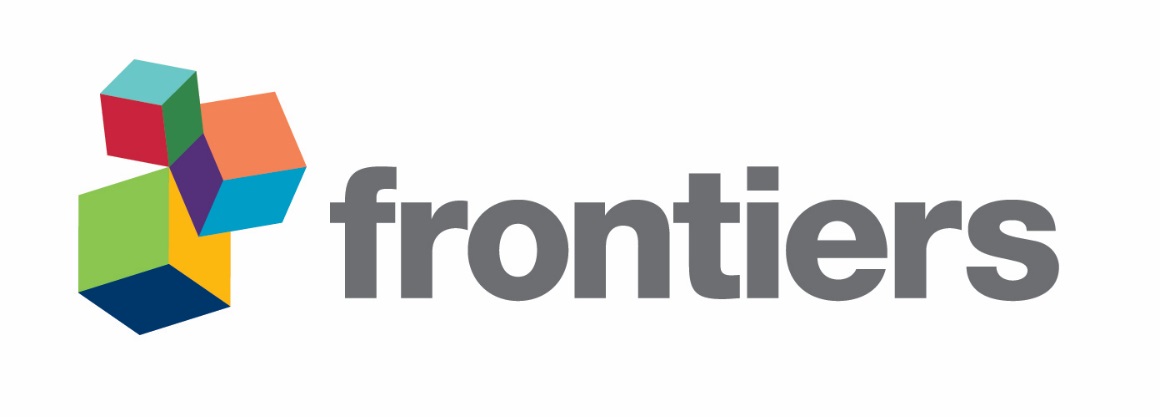
**
